# Mixed Matrix Carbon Molecular Sieve and Alumina (CMS-Al_2_O_3_) Membranes

**DOI:** 10.1038/srep30703

**Published:** 2016-07-29

**Authors:** Yingjun Song, David K. Wang, Greg Birkett, Wayde Martens, Mikel C. Duke, Simon Smart, João C. Diniz da Costa

**Affiliations:** 1 The University of Queensland, FIM2Lab – Functional Interfacial Materials and Membranes Laboratory, School of Chemical Engineering, The University of Queensland, Brisbane Qld 4072, Australia; 2 The University of Queensland, School of Chemical Engineering, The University of Queensland, Brisbane Qld 4072, Australia; 3 Science and Engineering Faculty, Queensland University of Technology, Brisbane, Qld 4000, Australia; 4 Institute for Sustainability and Innovation, Victoria University, Werribee, Vic 3030, Australia

## Abstract

This work shows mixed matrix inorganic membranes prepared by the vacuum-assisted impregnation method, where phenolic resin precursors filled the pore of α-alumina substrates. Upon carbonisation, the phenolic resin decomposed into several fragments derived from the backbone of the resin matrix. The final stages of decomposition (>650 °C) led to a formation of carbon molecular sieve (CMS) structures, reaching the lowest average pore sizes of ~5 Å at carbonisation temperatures of 700 °C. The combination of vacuum-assisted impregnation and carbonisation led to the formation of mixed matrix of CMS and α-alumina particles (CMS-Al_2_O_3_) in a single membrane. These membranes were tested for pervaporative desalination and gave very high water fluxes of up to 25 kg m^−2^ h^−1^ for seawater (NaCl 3.5 wt%) at 75 °C. Salt rejection was also very high varying between 93–99% depending on temperature and feed salt concentration. Interestingly, the water fluxes remained almost constant and were not affected as feed salt concentration increased from 0.3, 1 and 3.5 wt%.

Access to potable water is one of the major challenges facing both developed and developing nations. A large fraction of water on our planet is saline, locked in oceans (96.5%) and groundwater (0.93%)^1^, whilst the supply of remaining fresh water sources is coming under increased pressure from growing urban populations, industrialization and climate change. Desalination, or the process of producing potable water from saline sources by removing dissolved salts and minerals, is crucial in meeting the potable water challenge. Technologies to desalinate sea water (total dissolved solids (TDS) ~3.5 wt%) and brackish waters (TDS < 1.0 wt%) on a large scale have been in place for more than 60 years. These include conventional thermal methods like multi-stage flash, multi effect distillation^2^,^3^; the current gold standard membrane-based reverse osmosis (RO)^4^,^5^,^6^ and the more niche techniques like membrane distillation (MD)^7^,^8^, pervaporation (PV) and electrodialysis^9^,^10^. Only conventional thermal and RO technologies have been deployed at large scales. For the niche technologies their utilisation depends on production volume, energy consumption and feed water characteristics. MD and PV for examples have found applications in aggressive and/or highly saline feed streams^8^ which cannot be processed via RO.

Although MD and PV membranes were initially based on polymeric materials, research into inorganic membranes for desalination applications has been gathering pace in recent years. In contrast to their polymeric counterparts, the pore size of most of the inorganic membranes studies has generally been in the ultramicroporous region (*d*_p_ < 2 nm). Many of these membranes operate as molecular sieves, allowing the diffusion of water (which has a kinetic diameter of 2.6 Å), and hindering the passage of the larger hydrated salt ions such as Cl^−^–H_2_O (6.64 Å) and Na^+^–H_2_O (7.16 Å)^11^,^12^. Therefore, these inorganic membranes technically operate as pervaporation (PV) membranes in desalination, as the membrane plays an integral role in separation, as opposed to traditional MD membranes which simply act as a non-selective porous barrier to liquid transport. Initial PV inorganic membranes delivered low water fluxes (below 2 kg m^−2^ h^−1^) and were mainly based on molecular sieving silica^13^,^14^ and zeolites^15^,^16^,^17^ membranes. Recent improvements were reported by preparing interlayer-free cobalt oxide silica membranes^18^ and slightly mesoporous titania^19^ which deliver water fluxes above 5 kg m^−2^ h^−1^ for processing seawater (NaCl 3.5 wt%) and brine (NaCl 7.5 wt%), respectively.

In this work, we propose a new concept of inorganic mixed matrix membrane contrary to the conventional organic/inorganic mixed phases. Mixed matrix membrane development has been based mainly on polymeric membranes containing an inorganic phase spread out homogeneously in the membrane matrix. The aim of the inorganic phase was to confer functionalities to the membrane beyond the properties of the polymeric materials^20^. The proposed new method starts with the vacuum-assisted impregnation of an organic phase in the pores of a porous inorganic substrate. Subsequently, the organic phase is carbonised in an inert atmosphere, thus forming carbon molecular sieve (CMS) structures within the pores of the alumina substrate. The use of carbon material for desalination (or deionization) applications has been gathering pace including carbonised templated silica thin films asymmetric membranes^21^, graphene/polymer composite membranes^22^, and carbon nanorods in capacitive deionisation^23^. An advantage of CMS materials is that a large number of polymeric precursors have been carbonised and investigated such as polyfurfuryl alchohol (PFA)^24^, polyimides^25^ and phenolic resins^26^,^27^,^28^. Nevertheless, CMS membranes have been prepared as single thin films or single phase hollow fibres generally deployed for gas separation testing in view of their molecular sieving structure.

Here we report a novel inorganic mixed matrix membrane (CMS-Al_2_O_3_) composed of CMS in an alumina matrix for desalination. A phenolic resin was selected as the precursor, which was incorporated into an alumina substrate by vacuum-assisted impregnation to form a mixed matrix. This method allows for a single cycle of substrate impregnation and the carbonisation of the organic phase. The evolution of carbonisation of the phenolic resin was fully characterised by TGA, FTIR and N_2_ adsorption. CMS-Al_2_O_3_ mixed matrix membranes were synthesised based on the most suitable CMS synthesis conditions and the membranes were subsequently tested for desalination by pervaporation.

## Results and Discussion

**Figure 1:**
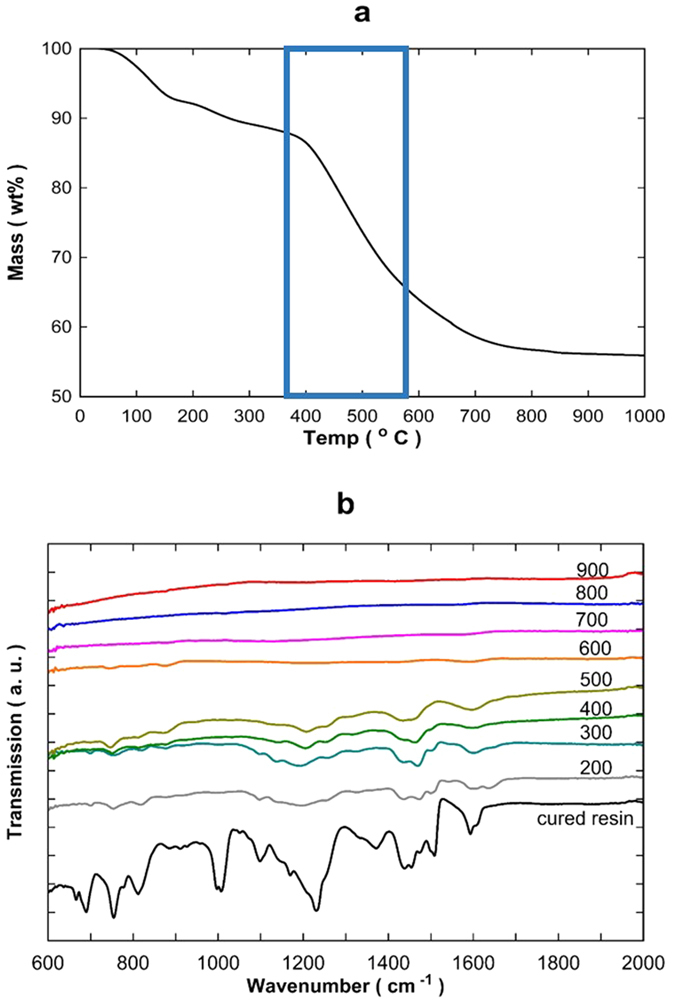
(**a**) TGA mass loss and (**b**) FTIR spectrum (fingerprint region) of cured and phenolic resin carbonized at temperatures from 200 °C to 900 °C.

TGA results in Fig. 1a display the thermal degradation behaviour of the cross-linked phenolic resin with hexamine and methanol under an inert atmosphere. The total mass loss reached 44.4% at 1000 °C, where 13.5% was measured up to 380 °C, followed by a major mass loss of 25.5% up to 650 °C, and 5.4% up to 1000 °C. The TGA-MS results (see Fig. S1 in Supplementary Information) suggest a complex decomposition generating several compounds. Water (18 amu), methylamine (31 amu) and dimethyl benzene (106 amu) contributed to the majority of the mass loss up to 380 °C which was attributed to desorption of the solvent and reaction by-products. Even though the CMS materials were dried in an oven for 24 h, water, methylamine and dimethyl benzene remained strongly adsorbed on the outer surface, particular inside the micropores, or was produced via further cross-linking. A major decomposition stage commenced near 380 °C and completed at 650 °C. This stage is characterized by several major decompositions of the phenyl derivatives from the backbone of the resin matrix, for example dimethanolamine (77 amu), benzene (78 amu), toluene (92 amu), phenol (94 amu), trimethyl benzene (120 amu), dimethyl benzene (106 amu) and methyl phenol (108 amu). The last stage of decomposition occurred above 650 °C where small molecular compounds such as water and CO_2_ were released. This mass loss was generally attributed to the further carbonization and dehydrogenation reaction of the aromatic rings forming polynuclear carbon structures^29^,^30^. The mass loss became insignificant for temperatures above 800 °C.

The chemical evolution of the carbonized resin as a function of temperature was also investigated by FTIR analysis as shown in Fig. 1b. Table S1 in the Supplementary Information lists the respective peak assignments^31^. The FTIR spectrum obtained from the cured phenolic resin (i.e. not carbonised) shows a large number of peaks overlapping in the lower wavenumber regions. The cluster of peaks in the fingerprint region of interest shows strong bands associated with hydrocarbons. These include the C=C stretch of phenyl rings (1600–1500 cm^−1^), the dominant peak assigned to C-O stretching of the phenols (1300–1000 cm^−1^), and several other small peaks associated with aromatic C-H bands. These FTIR results are in accordance with the representative spectrum of cured phenolic resins^32^,^33^. As the carbonization temperature reached 300 °C, peaks observed near 1380 and 1000 cm^−1^ which are associated with O-H and C-O stretching of the phenolic rings respectively, disappear. Further carbonization up to 500 °C did not show major changes in the fingerprint region indicating that the functional groups of the phenolic resin were retained to some degree. However, major changes occurred as the carbonization temperature increased from 500 to 600 °C, showing significant alterations in the phenolic resin spectra. These FTIR results are in line with the TGA-MS results, associated with measurements of the decomposition of phenyl derivatives from the backbone of the resin matrix. From 700 °C onwards, the bands in the lower wavenumber regions have almost disappeared indicating that there were no more functional groups (as detected by FTIR), thus suggesting that only carbon was retained as CMS materials, which constituted a 53% total weight.

**Figure 2:**
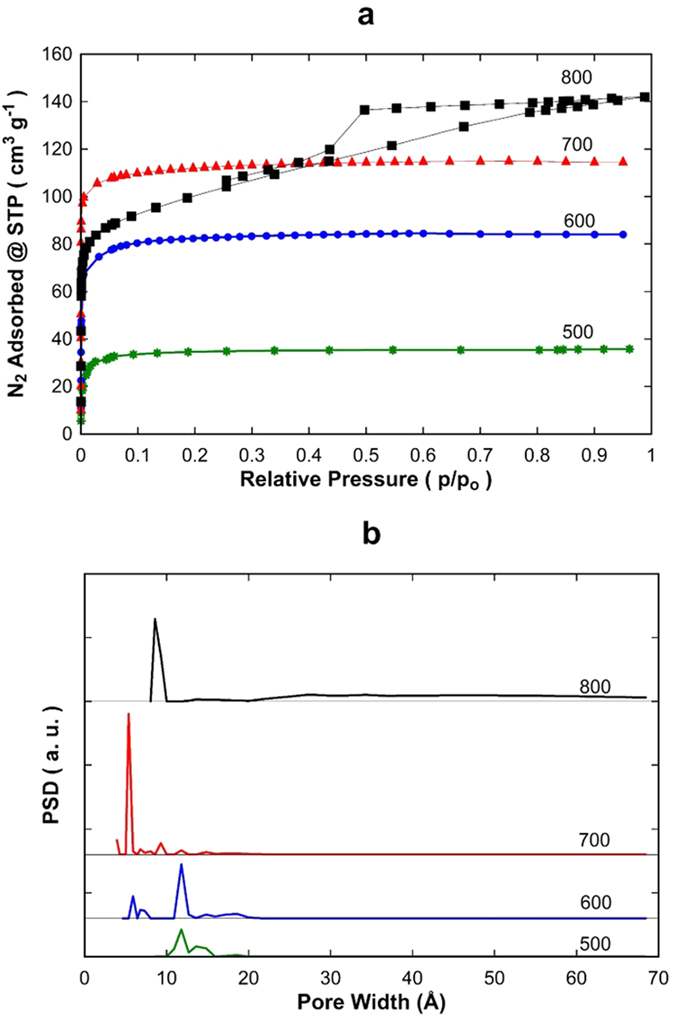
(**a**) Nitrogen adsorption isotherms and (**b**) PSD – pore size distribution for carbonised resins at temperatures from 500 to 800 °C.

Initial nitrogen adsorption showed that samples carbonized at temperatures below 500 °C were generally dense, displaying low surface areas. Figure 2a shows that the adsorption isotherms of CMS samples carbonised at 500, 600 and 700 °C, which are classified as type I isotherms and represent microporous structures. There is a rapid uptake of nitrogen adsorption at very low relative pressures (p/p_o_ < 0.05) followed by a constant saturation level. The sample CMS800 shows a type IV isotherm with a hysteresis loop at 0.4 < p/p_o_ < 0.9, which is a characteristic of mesoporous materials. Overall, the pore volume increased as the carbonization temperature was raised from 500 to 800 °C. Figure 2b shows the pore size distribution (PSD) as a function of carbonization temperature. Despite a sharp type I isotherm, the sample carbonized at 500 °C displayed 2 main peaks at pore widths of 12.5 and 14 Å. Increasing the carbonization temperature to 600 °C saw a trimodal PSD with the 12.5 Å retained, but new peaks at 6 Å and 18 Å can be observed. The lowest pore size of 5.5 Å and the narrowest distribution (additional small peaks at 9.5 Å and 12.5 Å) was seen at a carbonization temperature of 700 °C. Increasing the carbonisation temperature further to 800 °C shifted the PSD to having a microporous peak at 9.9 Å, and a broader mesoporous region between 20 and 70 Å.

The increase of pore volume is associated with the thermal decomposition of the phenolic resin as observed in the FTIR (Fig. 1b) and TGA-MS analysis (Fig. S1 Supplementary Information). As the phenol derivatives are decomposed together with the breakdown of linear hydrocarbons and other aromatics for the carbonisation temperatures from 500 and 600 °C, these functional groups leave the carbonizing matrix, thus conferring microporosity. This also includes the sample carbonised at 700 °C which is a transition between the major mass loss stage (380 to 650 °C) and the last stage (>650 °C). The sample carbonised at 800 °C is truly reaching into the last stage of carbonisation, and the mesoporosity evident in Fig. 2b are attributed to the carbonization and dehydrogenation reaction of the aromatic rings which in turn form polynuclear carbon structures as determined by TGA-MS. The BET surface area of the samples increased with temperature from 101 m^2^ g^−1^ (500 °C), to 254 m^2^ g^−1^ (600 °C) and 343 m^2^ g^−1^ (700 °C), then slightly reduced to 315 m^2^ g^−1^ (800 °C). The increase in surface area is directly related with microporosity formation during the carbonisation step, whilst the reduction at 800 °C is due to the loss of microporosity as the sample began to transform some of the microporosity into mesoporous structures.

**Figure 3:**
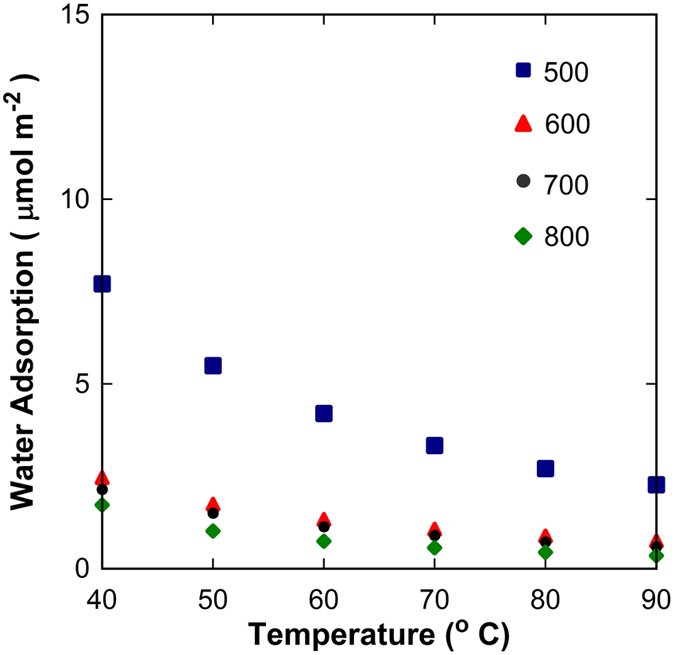
Equilibrium water adsorption per surface area at 3 kPa water vapour pressure as a function of temperature for carbonised resins at temperatures from 500 to 800 °C.

Carbon is well known to impart hydrophocity, though water adsorption is affected by both surface area and surface chemical properties^34^. Figure 3 shows the equilibrium water adsorption data as a function of temperature normalised by surface area. The sample carbonised at 500 °C has water adsorption one order of magnitude higher than that of the other samples at 40 °C. Hence, it appears the sample carbonised at 500 °C is significantly more hydrophilic than the other samples calcined at higher temperatures. The water adsorption coverage as a function of temperature for the samples carbonised at 600 to 800 °C is almost negligible thus indicating that the surface chemistry for these samples is likewise similar. These results strongly suggest the effect played by dangling hydroxyl groups, ether bonds among other aromatic structures, which are more prominent at carbonisation temperatures at 500 °C and tend to disappear at ≥600 °C, as discussed in the TGA-MS and FTIR analysis. These results are in line with what is commonly seen for heat treated carbons. As the carbonisation temperature increases the amount of functional groups decrease in both on non-porous^35^ and porous carbons^36^.

**Figure 4:**
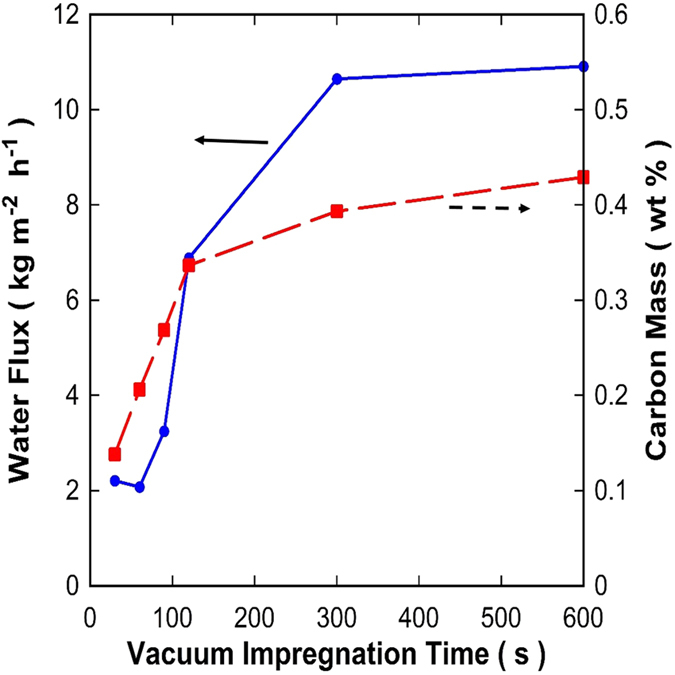
Water flux (±10%) (full line) at 25 °C and NaCl concentration of 0.3 wt%, and carbon mass (broken line) retained in the mixed matrix CMS-Al_2_O_3_ membranes carbonised at 600 °C as a function of the vacuum impregnation time. Permeate vacuum pressure <1 Torr.

Initially, the effect of vacuum impregnation time was investigated for the mixed matrix CMS-Al_2_O_3_ membranes carbonised at 600 °C. Figure 4 shows the water flux results tested for 0.3 wt% salt feed solutions at 25 °C. It is observed that the water fluxes increased as a function of the impregnation time and reached a plateau at 300 s. Short times of 30 and 60 s gave no major changes in water fluxes, which increased from 90 to 300 s. Membranes were also tested without vacuum impregnation (t = 0 s), though water fluxes were very low, around one order of magnitude below the values of ~2 kg m^−2^ h^−1^ for the membranes with vacuum impregnation times of 30 and 60 s. All the tested membranes delivered high salt rejections above 95%. TGA analysis of the membranes also displayed in Fig. 4 shows that the relative amount of carbon retained in the α-alumina substrate increased steeply from 30 to 120 s of vacuum time and then started stabilising with incremental amounts up to 600 s. Analysis of solvent collected in the cold trap prior to the vacuum pump by UV-Vis gave no readings for the phenolic resins. This clearly indicates that the solvent (methanol) permeated through the membrane, though the phenolic resin was retained in the α-alumina substrate during the vacuum assisted impregnation method.

**Figure 5:**
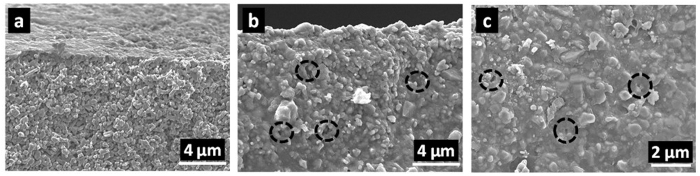
Representative SEM micrographs of mixed matrix CMS-Al_2_O_3_ membranes. (**a**) cross section of 30 s vacuum impregnation time, (**b**) cross section and (**c**) top layer of 600 s vacuum impregnation time. (Gaps are shown within broken line circles).

Further verification of the membrane cross sections by SEM shows the formation of a film on the top of the α-alumina substrate for the membrane prepared at 30 s vacuum impregnation time (Fig. 5a). At 600 s (Fig. 5b) there is no top film formation displaying a cross section fully impregnated with carbon whilst the top layer (Fig. 5c) shows homogeneous coverage of impregnated carbon around α-alumina particles. These results strongly suggest that when the vacuum time is short, for example 30–60 s, there is insufficient time to draw the resin into the pores. In this case whilst the carbon content is relatively small, the effective membrane thickness is relatively large as all the carbon is agglomerated at the surface of the alumina support. This result explains the low water flux results for the short time vacuum-assisted impregnation, as the thickness of the CMS film provides a high resistance for water diffusion. In other words, an asymmetric membranes was formed with a CMS film on the top of an α-alumina substrate. As the vacuum time increases, more resin is drawn further into the α-alumina substrate distributing the resin and filling all the α-alumina substrate pores spaces. As the resin fills the pores spaces, subsequent carbonisation introduces preferential percolation pathways with lower resistance for water diffusion, substantiated by the water fluxes increasing by 500% to 10.9 kg m^−2^ h^−1^ as the vacuum-assisted impregnation time changes from 30 to 600 s.

**Figure 6:**
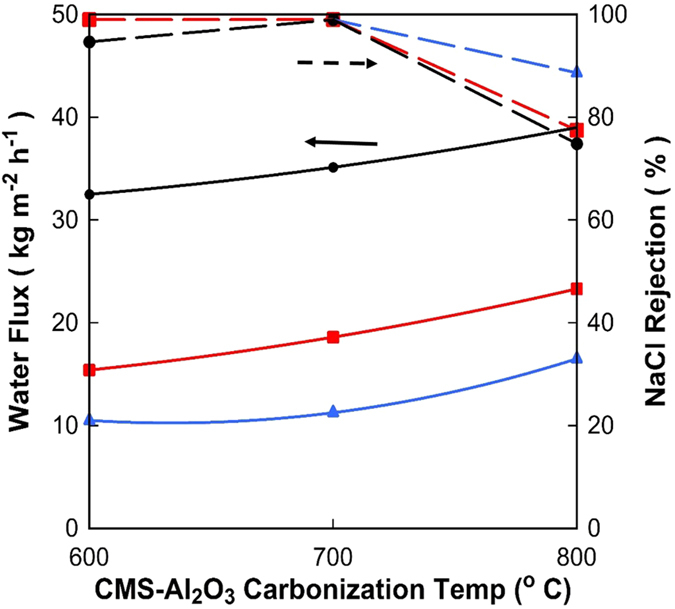
Water flux (±10%) (full line 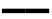) and salt rejection (±2%) (broken line 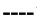) as a function of the carbonisation temperature for a NaCl 0.3 wt% concentration at varying temperatures (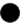 75, 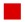 50 and 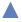 25 °C).

Subsequently, mixed matrix CMS-Al_2_O_3_ membranes prepared with a vacuum time of 600 s were carbonised from 600 to 800 °C and tested for 0.3 wt% salt feed solutions from 25 to 75 °C as shown in Fig. 6. Carbonisation at 500 °C was not considered due to the very low total pore volume (Fig. 2b) and small BET surface area. The water fluxes for all membranes were quite large, ranging from 17 to 40 kg m^−2^ h^−1^ and the trends were consistent with PV mechanisms with higher feed temperatures leading to increased water fluxes. The membranes carbonised at 800 °C resulted in the largest water fluxes, although the overall performance was compromised by a lower salt rejection between 75 and 88%. The salt rejection for the membranes carbonised at 600 and 700 °C was very high over 95% and ~99.8% for all feed solution temperatures, respectively.

The loss of separation performance for the membrane carbonised at 800 °C is directly related to the CMS mesoporous structures as seen in Fig. 2b, where the larger pore sizes 20 to 70 Å allowed for diffusion of the hydrated salt ions. In theory, salt rejection should remain at or close to 100% as salt is non-volatile. Even if it passes through the selective layer it should be dehydrated at the inner surface, remaining in the support as the water is vaporised by the vacuum. However, small droplets and even small salt crystals can become entrained in the vacuum flow and which are collected in the condensation trap. This has been observed before in several similar studies for inorganic asymmetric membranes containing molecular sieve silica structures^18^,^19^, zeolites^18^,^37^, polymeric mixed matrix membranes containing silica^38^ and carbon nanotubes^39^. Hence, the reduced salt rejection could be attributed to larger pores with a percolative pathway through the inorganic mixed matrix membrane. This demonstrates that the vacuum-assisted impregnation method is able to maintain the molecular sieving structures (seen in the carbonized powders) in the mixed matrix membranes. Further, the morphology of the CMS material in the alumina substrate probably has a very low concentration of percolative pathways with larger pores, thus hindering the permeation of hydrated salts.

**Figure 7:**
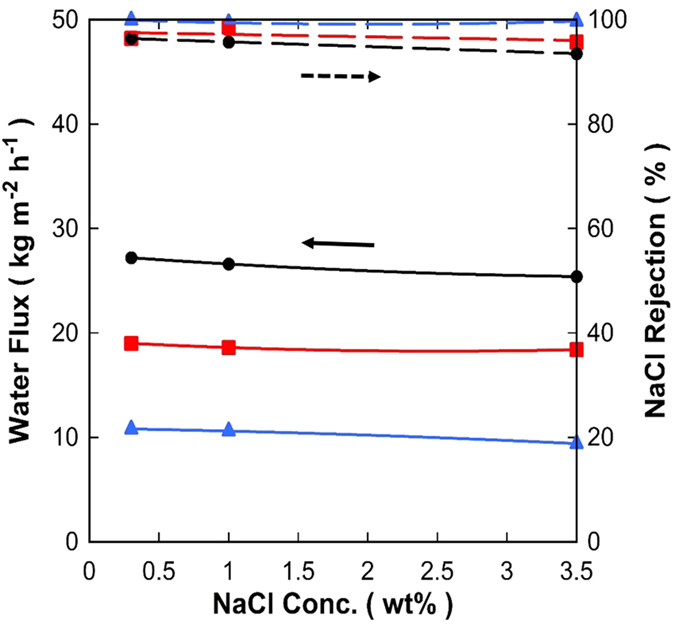
Water flux (±10%) (full line 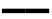) and salt rejection (±2%) (broken line 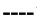) as a function of NaCl 0.3 wt% concentration at varying temperatures (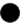 75, 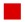 50 and 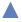 25 °C).

The membrane carbonised at 700 °C gave the best results in terms of water fluxes and salt rejection as observed in Fig. 6. Therefore, new batches of this membrane were prepared and further tested for varying feed salt concentration at brackish (0.3 and 1.0 wt%) and sea water (3.5 wt%) concentrations. Figure 7 shows high salt rejections (>93%) and that the water fluxes increased as a function of the feed temperature independently of the salt concentration. This is expected as the increase in feed water temperature corresponds to an increase in the vapour pressure at the interface between the membrane and the bulk liquid. As the permeate pressure was kept under constant vacuum (<1 torr), increasing the vapour pressure at the feed side generated a greater driving force, which is the vapour pressure gradient across the membrane, and likewise increased the water flux^40^,^41^.

It is interesting to observe in Fig. 7 that increasing the salt concentration in the feed water resulted in no significant change in water flux through the membrane. For example, in this work the feed concentration was raised from 0.3 to 3.5 wt%, which translates into vapour pressure change decrease of 1.6% to 1.8% for the feed stream at temperatures of 25 or 75 °C, respectively^42^. This reduction in driving force is less than the observed flux reduction of 5–10% depending on the feed stream temperature. Importantly, this variation in water fluxes lies within experimental error for the flux measurements. These results are in direct contrast to other studies using inorganic membranes with molecular sieving structures (silica and zeolite) for PV desalination^16^,^17^,^18^,^43^ which were strongly affected by feed salt concentration. Therefore, the disparity between the influences of salt concentration for different inorganic membranes is striking. The previous studies on silica based or zeolite membranes operated under similar conditions of a turbulent regime with a high Re number ~40000 as in this work. The explanation must therefore be associated with the membrane morphology and surface chemistry as demonstrated by de Lint *et al.*^44^ on the retention of Na^+^ and Cl^−^ ions on silica membranes coated on γ-alumina substrates. Presumably the more of these ions are retained on the surface of the membrane, the more likely they hinder water’s ability to migrate through which translates to lower flux. The results in this work strongly suggest that carbon structures may repel hydrated ions, or do not retain ions, thus reducing the concentration of salts on the membrane surface, contrary to the hydrophilic silica and zeolite base membranes.

The variation in flux and salt rejection for the membranes carbonised at 700 °C in Figs 6 and 7 is related to the morphology of different α-alumina substrates. This work used commercially available α-alumina tubes with porosities varying between 34.5 and 36.6%. The impregnation of the phenolic resin and subsequent carbonisation led to a relative reduction of porosity in region of 7.0% (e.g. from 36.6 to 34.1%). The overall performance of the inorganic mixed matrix CMS-Al_2_O_3_ membrane for processing seawater (NaCl ~3.5 wt%) is noteworthy. Water fluxes reaching values as high as 25 kg m^−2^ h^−1^ (at 75 °C), thus outperforming most of the inorganic PV membranes tested for desalination. For instance, molecular sieve silica base^18^, mesoporous carbon-silica^45^ and organo-silica^24^ membranes in a span time of 10 years have improved water fluxes from ~1 to 11 kg m^−2^ h^−1^, respectively, while best zeolite PV membranes^46^ delivered values up to 5 kg m^−2^ h^−1^. Although hybrid polymer-inorganic membranes based on poly(vinyl alcohol)/maleic acid/silica^47^ gave lower water fluxes ~7 kg m^−2^ h^−1^ (at room temperature), recent graphene oxide/polyacrylonitrile (GO-PAN)^48^ membranes produced higher values of ~46 kg m^−2^ h^−1^ at 70 °C. The GO-PAN is very thin ~1 μm, 300 times thinner than the CMS-Al_2_O_3_ membrane in this work. As the water flux is inversely proportional to the thickness of the membranes, this work opens a window of opportunities to reduce the thickness of inorganic mixed matrix CMS-Al_2_O_3_ membrane for processing seawater and deliver very high output potable water production.

**Figure 8:**
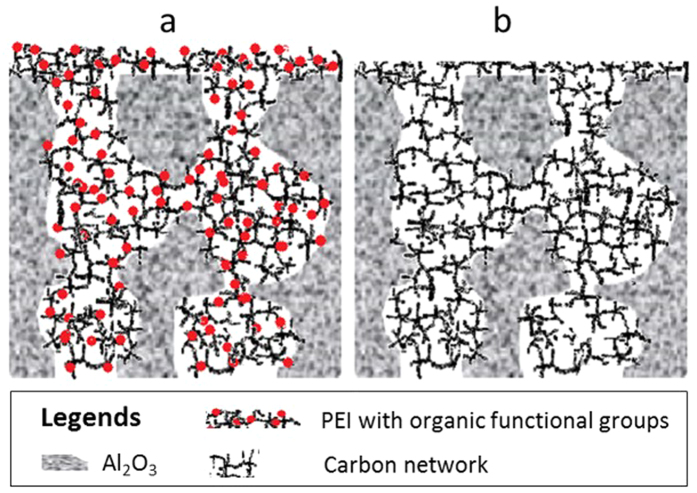
Schematic of mixed matrix CMS-Al_2_O_3_ membranes. (**a**) after vacuum-assisted impregnation and (**b**) after carbonization.

The SEM micrographs in Fig. 5b,c clearly show that the resin was able to penetrate into the pores of the α-alumina substrate by the vacuum-assisted impregnation method. The α-alumina particles appear to be fully coated by carbon upon the carbonisation of the resin. A few minor gaps are observed, which could be related to the vacuum-assisted impregnation method, or due to small fragments when the membrane was broken for SEM analysis. Nevertheless, there does not appear to be any connection between the gaps, which is supported by the high salt rejection values in Fig. 6. A schematic of the mixed matrix CMS-Al_2_O_3_ membrane is displayed in Fig. 8 where it is postulated that the vacuum-assisted impregnation of phenolic resin caused pore filling in the α-alumina substrate. In this process, the phenolic resin was entrained in the methanol solvent, which permeated through the porous structure of the substrate. Short-term vacuum permitted the formation of a thick top layer on the substrate as confirmed by the SEM image in Fig. 5a, thus adding a higher resistance to water transport as evidenced by the low water flux in Fig. 4. With longer vacuum time, the top layer was further broken down by the solvent and the resin permeated deeper into the substrate. As a result, the total amount of the resin impregnates into the porous structure increased (Fig. 4), though it is interesting to note that the resistance to water transport reduced as water fluxes increased (Fig. 4). These results strongly suggest that short-term vacuum formed denser films, in contrast to long vacuum times produced less compact structures with increased porosity whilst maintaining a narrow pore size required for high salt rejection. Upon carbonization, the organic functional groups of the resin were decomposed in an inert atmosphere at high temperature above 600 °C. Consequently, the CMS structures were formed in the pores of the substrate and around the α-alumina particles, resulting in a mixed matrix CMS-Al_2_O_3_ membrane. This CMS structure was responsible for controlling the performance of the CMS-Al_2_O_3_ membrane in terms of water fluxes and salt rejections.

In summary, the penetration of phenolic resins into α-alumina substrates resulted in pore filling via a vacuum-assisted impregnation method. Upon carbonisation, the phenolic resin decomposed with an initial mass loss due to strongly adsorbed methanol solvent, followed by significant mass loss between 400 and 600 °C related to the decomposition of phenyl derivatives from the backbone of the resin matrix including benzene, toluene and phenol. The final decomposition stage at temperatures >650 °C were associated with a minor mass loss and the formation of carbon structures with molecular sieving dimensions (e.g. CMS). Best CMS structures with the lowest average pore sizes of ~5 Å were obtained at carbonisation temperatures of 700 °C, though higher carbonisation temperature at 800 °C led to the undesirable formation of mesoporous structures, whilst dense structures were observed for temperatures <500 °C. Prepared membranes resulted in a mixed matrix of α-alumina particles and CMS structures (CMS-Al_2_O_3_). These membranes were tested for desalination application and using best 700 °C carbonisation temperature delivered the best performance in terms of water fluxes and salt rejection. Best membranes resulted in very high water fluxes up to 25 kg m^−2^ h^−1^ for seawater (NaCl 3.5 wt%) in a pervaporation system at 75 °C, whilst maintaining salt rejections in excess of 93%. It is noteworthy that the water fluxes of mixed matrix CMS-Al_2_O_3_ membranes were almost constant for all temperature tested as the salt concentration increased from 0.3 to 1 and 3.5 wt%. Hence, concentration polarisation was not significant. This result was attributed to the carbon structure, which imparts hydrophobicity, contrary to previous hydrophilic inorganic membranes.

## Methods

### Materials

A phenolic resin Novolak Resinox IV-1058 (Orica Chemicals) was diluted with methanol before cross linking was initiated with hexamine. The mixture was stirred continuously for 20 min, then left to react for 2 h at room temperature before being dried in air for 24 h and then dried in vacuum for another 24 h to remove any remaining solvents. The dried and cured phenolic resin was carbonized in a quartz tube in a furnace using a nitrogen atmosphere. Carbonisation was carried out at desired temperatures up to 900 °C with a dwell time of 1 h and a heating rate of 5 °C min^−1^.

Thermogravimetric analysis (TGA) was performed on a Shimadzu TGA-50 under high-purity nitrogen flowing at 80 ml min^−1^ from 25 °C to 1000 °C. More detailed thermal analysis in the form of thermogravimetric analysis coupled mass spectrometry (TGA-MS) was used to study the decomposition of phenolic resin with temperature. The TGA-MS consisted of a high resolution TGA from TA instruments (series Q500) coupled to a Thermostar (Pfeiffer) mass spectrometer for gas analysis. Fourier Transform Infrared (FT-IR) analysis was conducted on a Shimadzu IR Affinity-1 FT-IR analyser with an ATR attachment over a range of 4000–400 cm^−1^. Nitrogen adsorption experiments were carried out on an ASAP 2020 System (Micromeritics Instrument Corporation), using the volumetric method to calculate the adsorbed amount of nitrogen. The samples were degassed at 200 °C for 24 h under high vacuum.

Water adsorption at ambient temperature was conducted on a custom-built gravimetric rig. Prior to the measurements, the samples were degassed under vacuum at 200 °C overnight. Water vapour isotherms were obtained by monitoring the sample weight change relating to water adsorption measured at increasing pressure increments by a high precision pressure transducer until the water vapour saturation pressure was attained. The mass change was calculated from the displacement of a quartz spring which was monitored by a camera. Water adsorption was also conducted by using a Shimadzu TGA-50. The CMS samples were degassed firstly under nitrogen atmosphere at 300 °C for 1 h. Then the samples were exposed to water vapour carried by pass feed nitrogen through a gas bubbler and held for 1 h at each pre-set temperature, decreasing from 100 to 20 °C in 10 °C increments with a cooling rate of 5 °C min^−1^. The gas bubbler and gas feed line into the TGA were kept at a constant temperature of 90 °C using a hot plate and heating tape, respectively.

### Membranes

Coating solutions were prepared by mixing the phenolic resin Novolak Resinox IV-1058 with an appropriate amount of hexamine to initiate cross linking before being diluted with methanol to form a 1:99 wt% coating solution. The fresh phenolic resin was vacuum impregnated via a dip-coating method. The outer shell of an α-alumina substrate tube (OD = 9 mm, ID = 6 mm, L = 525 mm, Ceramic Oxide Fabricates, Australia) was inserted in a beaker containing a phenolic resin. A three -way valve was opened to a vacuum line with pressures below 1 Torr. This allowed for the phenolic resin to impregnate the pores of the α-alumina substrate tube. Upon reaching required time of vaccum impregnation, ranging from 30 to 600 s, the three-way valve was closed and opened to atmosphere to depressurise the inner shell of the membrane. Subsequently, the vacuum-assisted impregnated membranes were air dried for 24 h, followed by vacuum-drying for another 24 h. Finally, the dried phenolic resin membranes were carbonized at a desired temperature for 1 h under nitrogen atmosphere with a heating and cooling rate of 5 °C min^−1^.

The solution collected in the cold trap at 77 K (i.e. liquid nitrogen) was analysed using a UV-Vis spectrophotometer Evolution 220 Thermo Fisher Scientific. The porosity of substrate tubes and the vacuum-assisted impregnated membranes after carbonisation were measured by helium pycnometry with a AccuPyc 1340 gas pycnometer. The surface morphological features of the mixed matrix CMS-Al_2_O_3_ membranes were examined using a JEOL JSM-7001F scanning electron microscope (SEM) with an accelerating voltage of 10 KV. The membranes were broken with segments used for cross-section and surface measurements. The segments were mounted on SEM stubs and platinum coated using a Baltec coating apparatus in high purity argon.

The membranes were tested for desalination using a pervaporation setup as described elsewhere^43^. Briefly, a tubular CMS membrane was immersed into a large beaker of feed salt solution which was placed on a hotplate stirrer. One end of the tube was blocked and the other side was connected to a vacuum pump via a cold trap. The outer shell of the mixed matrix CMS-Al_2_O_3_ membrane was at direct contact with the feed solution under atmospheric pressure. The inner shell was under vacuum conditions, thus creating the driving force for vapour to permeate though the membrane and to be collected in the cold trap. The water flux was calculate as *J* = *m/(A.t)*, where *J* (kg m^−2^ h^−1^) is the flux, *m* (kg) is the mass of water from the cold trap, *A* is the membrane surface area (m^2^), and *t* (h) is the time over a testing period. Although a separation factor is used in pervaporation of liquid/vapours, in desalination salt rejection is used to determine the separation property of the membranes. The salt rejection was calculated as *R* = *(C*_*f*_ − *C*_*r*_)/*C*_*f*_ × 100% based on conductivity of solutions, where R(%) is the salt rejection, *C*_*f*_ and *C*_*p*_ are the salt concentrations (NaCl wt%) in the feed and permeate streams, respectively. The salt concentration was calculated from conductivity measurements using a conductivity meter LabCHEM CP. The feed solution was stirred constantly, and also recirculated in the large beaker with the assistance of a peristaltic pump. The Reynold’s number of the membrane in the stirred beaker was in the turbulent range, calculated based on the vortex^49^ generated by the fast stirrer, and generating approximately 2–3 × 10^4^. The feed temperature was controlled by the thermostatically-controlled hotplate stirrer and was varied in the range of 20–75 °C. Feed solutions with concentrations ranging from 1 to 3.5 wt% were obtained by dissolving an appropriate amount of NaCl (Sigma Aldrich) into deionised water.

## Additional Information

**How to cite this article**: Song, Y. *et al.* Mixed Matrix Carbon Molecular Sieve and Alumina (CMS-Al_2_O_3_) Membranes. *Sci. Rep.* **6**, 30703; doi: 10.1038/srep30703 (2016).

## Supplementary Material

Supplementary Information
