# Supplementary material for: Mixed Matrix Carbon Molecular Sieve and Alumina (CMS-Al2O3) Membranes
Source: Sci Rep. 2016 Jul 29;6:30703. doi: 10.1038/srep30703 (PMC4965814; doi:10.1038/srep30703)
Supplement: Supplementary Information [file srep30703-s1.doc]

**Mixed Matrix Carbon Molecular Sieve and Alumina (CMS-Al_2_O_3_) Membranes**

Yingjun Song,^1^ David K. Wang,^1^ Greg Birkett,^2^ Wayde Martens,^3^ Mikel C. Duke,^4^ Simon Smart^1^ and João C. Diniz da Costa^1*^

^1^The University of Queensland, ^1^FIM^2^Lab – Functional Interfacial Materials and Membranes Laboratory, ^1,2^School of Chemical Engineering, The University of Queensland, Brisbane Qld 4072, Australia.

^2^The University of Queensland, School of Chemical Engineering, The University of Queensland, Brisbane Qld 4072, Australia.

^3^Science and Engineering Faculty, Queensland University of Technology, Brisbane, Qld 4000, Australia.

^4^Institute for Sustainability and Innovation, Victoria University, Werribee, Vic 3030, Australia

**Supplementary Information**


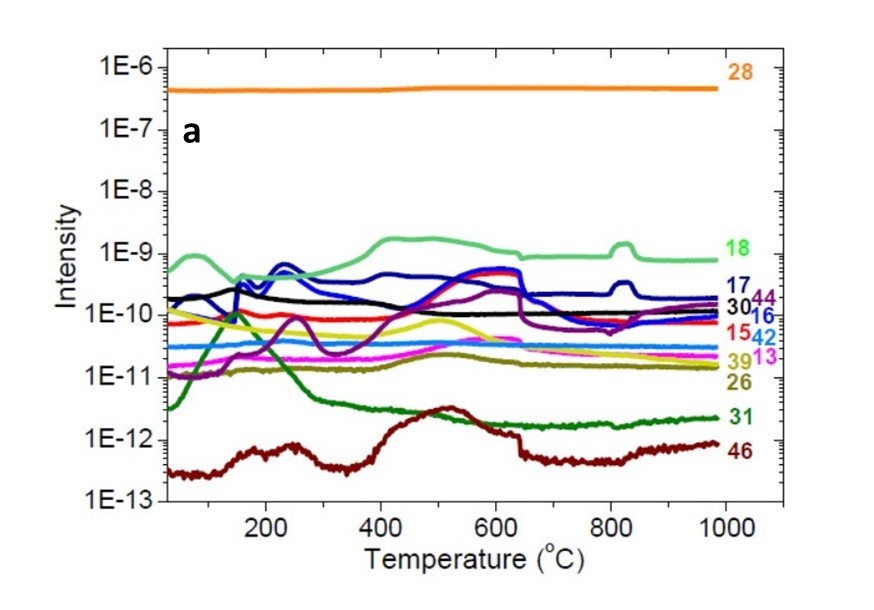

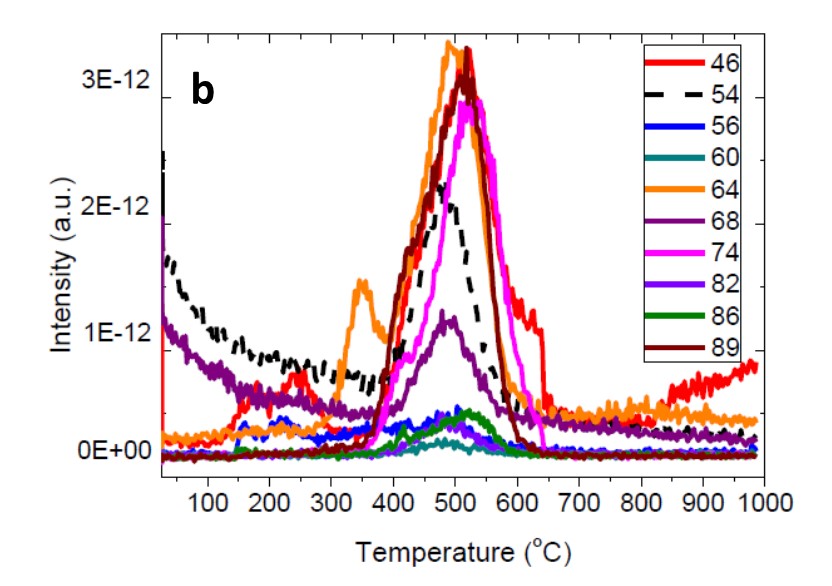


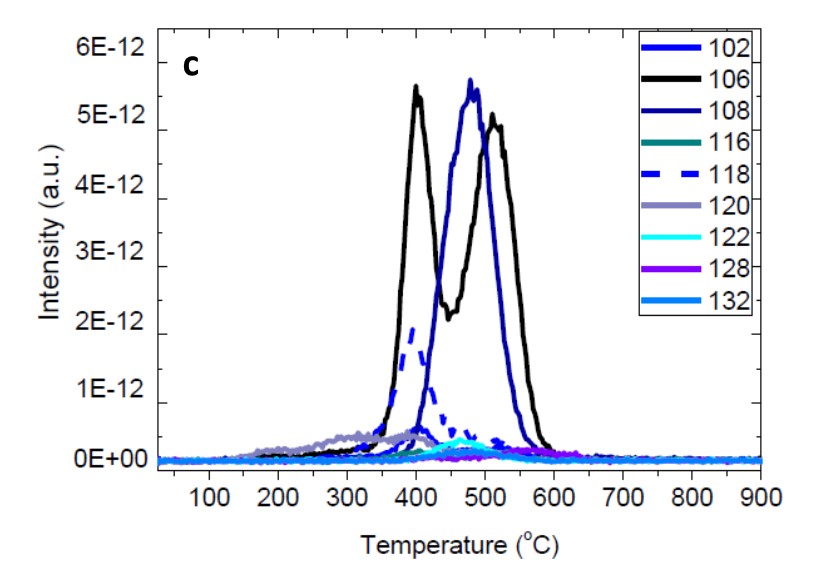


Fig. S1 – MS-TGA of the decomposition of the phenolic resin (a) range of 16-46 amu, (b) range of 49-89 amu, and (c) range of 102-132 amu.

Table S1 Vibrational assignment of phenolic resin [1].

| Region | Peaks | Functional group |
| --- | --- | --- |
| 1600-1450 C=C ring stretching (1600, 1580, 1500, 1450 for standard) | 1609 | C=C ring stretching components indicate 1,2,4- disubstitution; |
|  | 1593 | 1593 benzene ring double bond C=C |
|  | 1592 | II ring stretching component 1,2,6 –disubstitution |
|  | 1510 | stong sharp III ring stretching component1,4- or 1,2,4- disubstitution |
| 1500-1370 aliphatic deformation vibration; | 1500 |  |
| 1465-1340 C-H bending of aliphatic bridge structure | 1478 | 1475 C-H bending of aliphatic bridge structure |
|  | 1456 |  |
|  | 1439 | 1440 C-H bending of Aliphatic ether |
|  | 1377 | Phenolic OH in-plane deformation |
| 1300-1000 C-O stretching of Phenol | 1340 | 1340, aromatic CO stretch |
| 1270-1230 C-O stretching of diphenyl ether(Ar- O) | 1254, | CO typical of alky-phenols |
|  | 1230 | C-O stretching of dipheyl ether structure |
|  | 1214 | CO 1200 Diphenylene ether; |
|  | 1169 | stretching of phenol group |
|  | 1148 | 1110, Aromatic in-plane CH def. |
| 1050-1000 CO of RO | 1113 |  |
|  | 1098 | 1110, Aromatic in-plane CH def |
|  | 1009 | Unreliable aliphatic CH2 wag |
|  | 995 | Unreliable aliphatic CH2 wag |
| 880-680 CH out of plane vibration varied with substitution | 889 |  |
|  | 812 | Aromatic C-H wagging modes: strong band near 820cm-1 for 1,4 and 1,2,4 substitutions |
|  | 776 | Aromatic C-H wagging modes: 780-740 (strong )1,2 and 1,2,6 substitution |
|  | 755 | 688-754 aromatic C-H bending fingerprint |
| OH out of plane vibration of phenol | 666 |  |

Reference

1. G. Socrates, Infrared and Raman characteristics group frequencies: Tables and charts, 2001 John Wiley & Sons Ltd., West Sussex.
